# Robotic CME in obese patients: advantage of robotic ultrasound scan for vascular dissection

**DOI:** 10.1007/s11701-022-01398-6

**Published:** 2022-04-15

**Authors:** Vicky Maertens, Samuel Stefan, Ioannis Mykoniatis, Najaf Siddiqi, Gerald David, Jim S. Khan

**Affiliations:** 1 Department of Colorectal Surgery, Portsmouth Hospitals University, NHS Trust, Portsmouth, UK; 2 University Hospital Dorset NHS Foundation Trust, Poole, Dorset, UK; 3 Faculty of Sciences, School of Health Sciences and Social Work, University of Portsmouth, Portsmouth, UK; 4 Queen Alexandra Hospital, Southwick Hill Road, Cosham, Portsmouth, PO6 3LY UK

**Keywords:** Robotic colorectal surgery, Complete mesocolic excision, Robotic ultrasound scan, Obese patients

## Abstract

Complete mesocolic excision (CME) in right-sided colon cancers appears to confer oncological benefits compared to conventional colectomy. Identification of the superior mesenteric vein (SMV) remains challenging. We describe the novel use of intra-operative robotic ultrasound scan (rUSS) in obese patients (BMI ≥ 29). All consecutive patients having robotic CME for colon cancer between 2014 and 2017 were included in this retrospective cohort study. Data were recorded on an ethics approved prospective database and included patient demographics, clinical and oncological outcomes. Patients were divided into group 1 (BMI ≤ 28) and group 2 (BMI ≥ 29). SMV first approach was employed in all cases and SMV detection was aided using rUSS in group 2. Primary outcome was postoperative morbidity. Secondary outcomes included conversion rate, operative time and length of stay (LOS). 41 (group 1, median 66 years) were compared to 32 patients (group 2, median 63 years). There were no conversions to laparoscopy or laparotomy. Median operative times for group 2 were 30 min longer (186 vs. 216 min, *p* = 0.05). Overall morbidity was similar (20% vs. 19% in group 1 and 2, *p* = 0.26). There was no significant difference between the two groups with regard to LOS (median 7 vs. 6 days, *p* = 0.48), readmissions (2 vs. 5, *p* = 0.13), R0 resection rate (98% vs. 94%, *p* = 0.43) and lymph node harvest (median 31 vs. 30, *p* = 0.28).CME can be technically more challenging than conventional colectomy in obese patients and is associated with longer operative times. The use of rUSS in obese patients can help to identify SMV and allow safer dissection.

**Supplementary Information:**

The online version contains supplementary material available at 10.1007/s11701-022-01398-6.

## Introduction

Complete mesocolic excision (CME) in patients with right sided colon cancer has been shown to have causal relationship with improved oncological outcomes compared to conventional colectomy. CME is associated with better 5-year overall survival rate and lower recurrence rate than non-CME surgery [1, 2]. Favourable outcomes of open CME have been replicated with laparoscopic and robotic approaches and the latter can be beneficial in comparison with laparoscopic technique for performing intracorporeal anastomosis and improved dissection as evidenced by lower conversion rates.[3, 4]

Improved outcomes with CME can be achieved by en bloc removal of the malignant lesion with increased amount of the colonic mesentery with careful dissection through the proper plane of mesocolic excision and central vascular ligation at the root of the vessels [2]. CME, however, has been perceived to be associated with a higher postoperative complication risk and greater intra-operative blood loss than conventional colectomy [5]. It is a more complex and technically demanding operation due to variability in vascular anatomy and the associated potential risk of vascular injury [3]. Damage to the superior mesenteric vein (SMV) is a serious intra-operative complication during CME, resulting in prolonged ileus or venous colonic ischemia. This damage can occur due to misinterpretation of the anatomy, variations in anatomy, diathermy or traction injuries or due to excessive retraction during the dissection of the middle colic trunk [6].

While CME surgery is easier in low BMI patients with thin mesentery making the visualisations of the key vascular structures easier, the same cannot be said about patients with high BMI. In obese patients, the visibility of vascular anatomy can be compromised by the amount of intra-abdominal adipose tissue.

In 2019, a review was published describing tips and tricks to overcome challenges in performing robotic gynaecologic surgery in obese patients [7]; however, literature concerning tips and tricks overcoming challenges in robotic colorectal surgery in obese patients is limited. In 2019 and 2020, a meta-analysis and review appeared describing the impact of robotic colorectal surgery in obese patients; however, they did not describe any techniques to facilitate the procedure in obese patients [8, 9]. To avoid morbidity caused by SMV injury during CME surgery, identification of SMV in obese patients can be facilitated with the novel use of intraoperative ultrasound scan as reported by our institution previously [10]. This paper is the first to present the outcomes of novel use of robotic ultrasound for SMV identification.

The primary aim of this study was to evaluate postoperative complications and clinical outcomes after robotic CME for right sided colon cancer, comparing patients in group 1 with low BMI (28 or less) and group 2 with high BMI (29 or more) where SMV visualisation was aided by the use of rUSS.

## Methods

All consecutive patients who underwent elective surgery for right-sided colon cancer with curative intent between December 2014 and December 2017 at our institution were included in this retrospective cohort study if they either had locally advanced T3/T4 tumours or N1/N2 disease on the preoperative CT. Right sides colon cancer was defined as tumours located in the ascending colon or the proximal third of the transverse colon. Emergency operations for perforation or obstruction and surgeries performed in patients with metastatic disease were excluded. Data were kept on a prospectively maintained colorectal cancer database.

Resections were carried out using the da Vinci X ® (Intuitive Surgical, USA) 4th generation system.

Patients were divided into group 1 (non-obese, BMI ≤ 28) and group 2 (obese, BMI ≥ 29). The use of robotic ultrasound probe was standard procedure in obese patients for safer identification of SMV.

The robotic team at Portsmouth Hospitals NHS Trust, UK is very experienced and has performed over 2000 robotic procedures to date. The unit performs on average 120 complex robotic colorectal resections per year including 40 robotic CME procedures using the Da Vinci X® system. A fellowship-training program has been in place for over 10 years with robotic TME fellowship offered using a standardised technique in TME surgery. Robotic CME with suprapubic approach was practiced in cadaver models in 2015 and over a period of 5 years the technique was refined to a standardised procedure for surgical training.

Patient demographics, tumour characteristics, operative findings and postoperative outcomes, including conversion rate, operation time, LOS, complication rate and readmissions, were recorded. Postoperative complications occurring within 30 days of the operation included bleeding, ileus, anastomotic leakage, intra-abdominal abscess and wound infections. In addition, oncological results such as lymph node harvest (LNH), lymph node ratio (positive lymph nodes/lymph nodes harvest; LNR) and percentage of clear pathologic margins were compared between the 2 groups.

Statistical analysis was carried out using the software package IBM SPSS v26. The significance level was defined at *α* = 0.05 and statistical tests were two-sided. The values for the different groups deviated significantly from the Gaussian distribution (*p* < 0.05); therefore, nonparametric tests were used for all statistical analyses. Mann–Whitney *U* test was used to compare both groups.

### Surgical technique

**Fig. 1:**
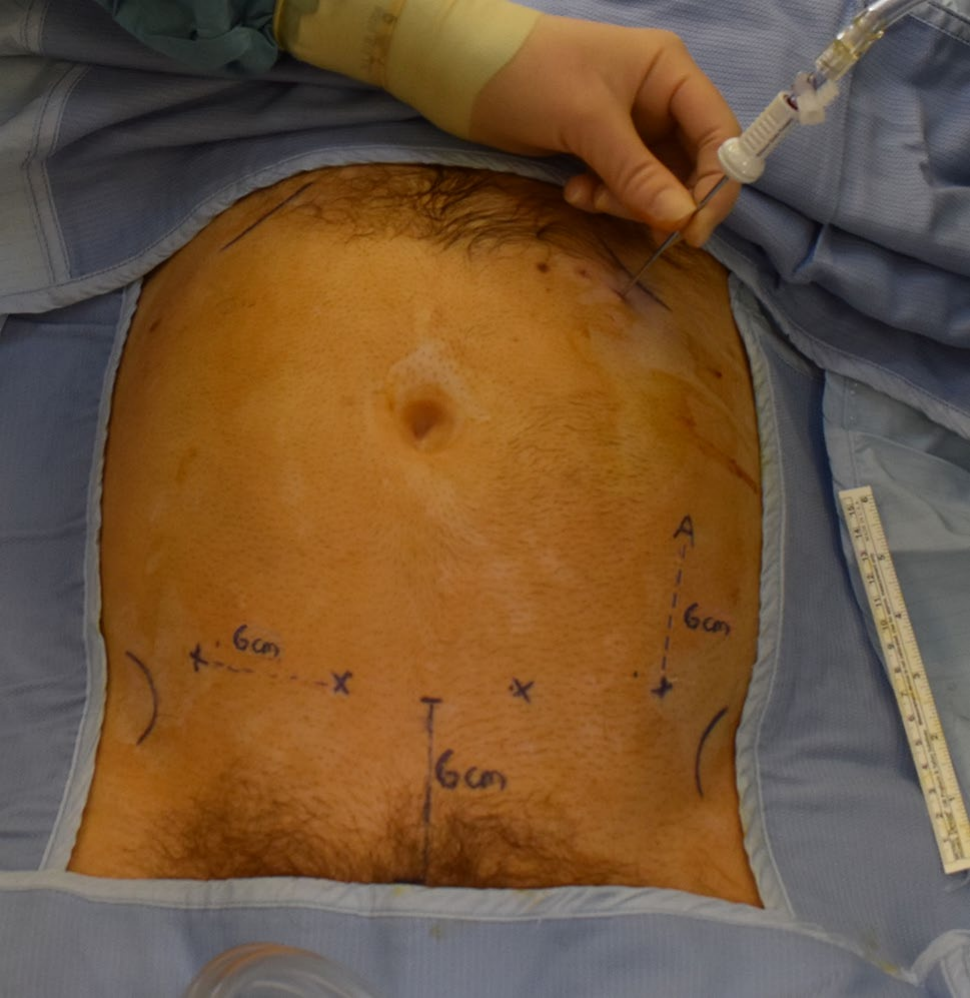
Suprapubic port placement 6 cm above the pubic bone. The X marks the 4 robotic ports and A marks the assistant port

**Fig. 2:**
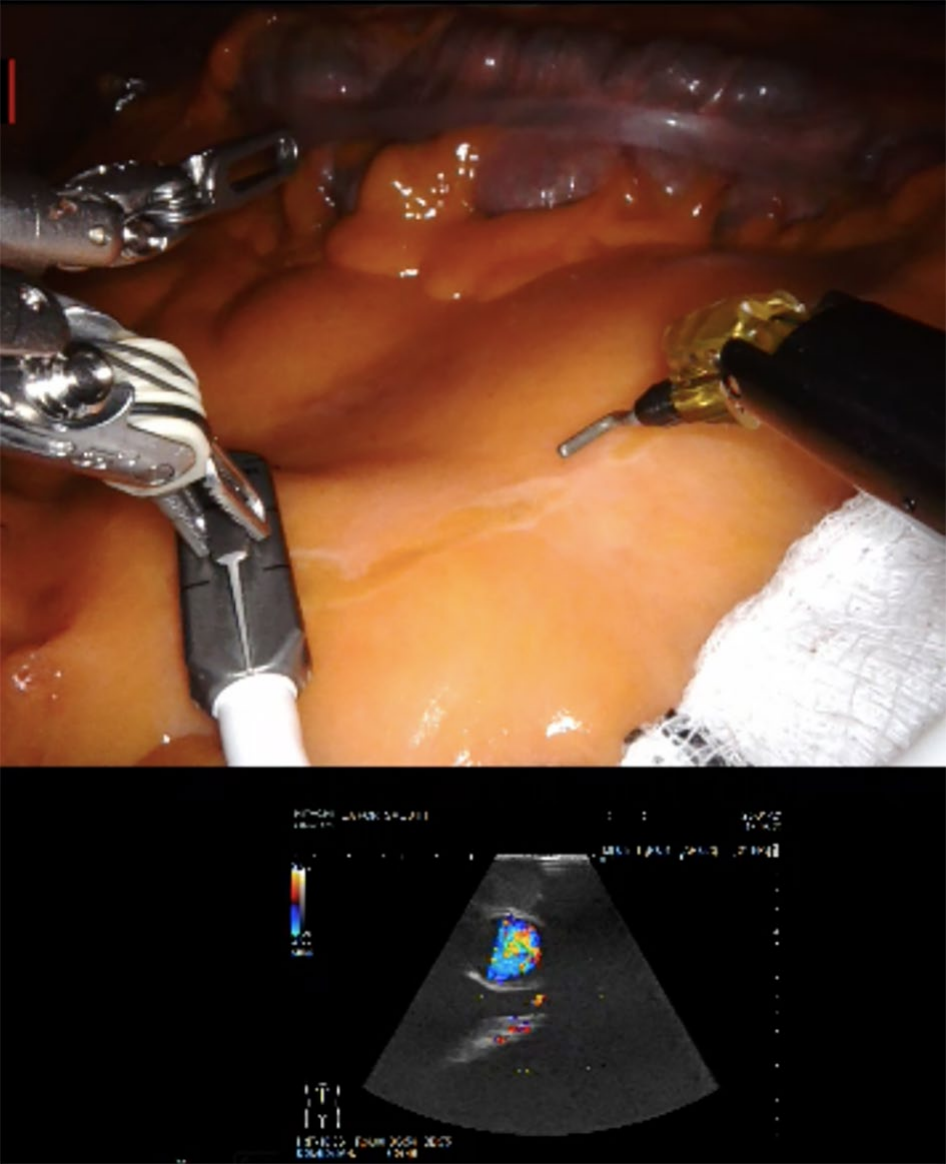
The ultrasound view in TilePro TM vision at the surgeon’s console and marking of the SMV as identified by the ultrasound device using monopolar diathermy with hook

In all patients, a suprapubic robotic port placement is caried out (Fig. 1). The first step of the procedure is dissection along the SMV, which is facilitated by the use of an ultrasound probe in obese patients. The robotic linear ultrasound probe (Hitachi, L51K Arietta) is placed through a 10 mm port and controlled robotically to identify the SMV [10]. The ultrasound view is seen in TilePro™ vision at the surgeon’s console and monopolar diathermy with hook is used to mark the line of SMV as identified by the ultrasound device (Fig. 2). An incision is made and the SMV is dissected along the indicated line with then ligation of the ileocolic vessels at their roots. Subsequently, Henle's trunk is identified, and the middle colic vein and right branch of the middle colic artery are ligated. Vessel dissection and ligation is illustrated in online animation (Online Resource 1). A medial-to-lateral dissection is carried out and a Robotic stapler SureForm™ 60 blue is used for transection of the transverse colon, terminal ileum and for the intracorporeal anastomosis formation. There is a subsequent suprapubic extraction of the specimen by Pfannenstiel incision. Anastomotic vascular perfusion is evaluated using Indocyanine Green Angiography (IcGA) in all patients.

## Results

Thirty-two obese patients (group 2, mean age 63 ± 12.81 years) were compared with 41 non-obese patients (group 1 mean age 66 ± 15.28 years). Patient demographics and TNM classification for each group are listed in Table 1. No statistically significant differences were found in age, gender and ASA grade between the groups (*p* = 0.44, *p* = 0.35, *p* = 0.54 respectively). Similarly, no significant difference was found in tumor stage and previous abdominal surgery (*p* = 0.68).

**Table 1:** Demographics and tumor characteristics

Parameter	Group 1 (*N* = 41)	Group 2 (*N* = 32)	*p* value
Age, years, mean (min–max)	66 (34–89)	63 (39–82)	0.44
Gender, *n* (M:F)	16:25	16:16	0.35
ASA (I/II/III)	7/25/9	5/23/4	0.54
Previous abdominal surgery (%)	16 (39%)	11 (34%)	0.68
cTNM			
T (1/2/3/4)	0/11/27/2	1/10/19/2	0.67
N (0/1/2)	11/22/8	16/13/3	0.11
M (0/1)	37/4	30/2	0.59

### Clinical outcomes

**Fig. 3:**
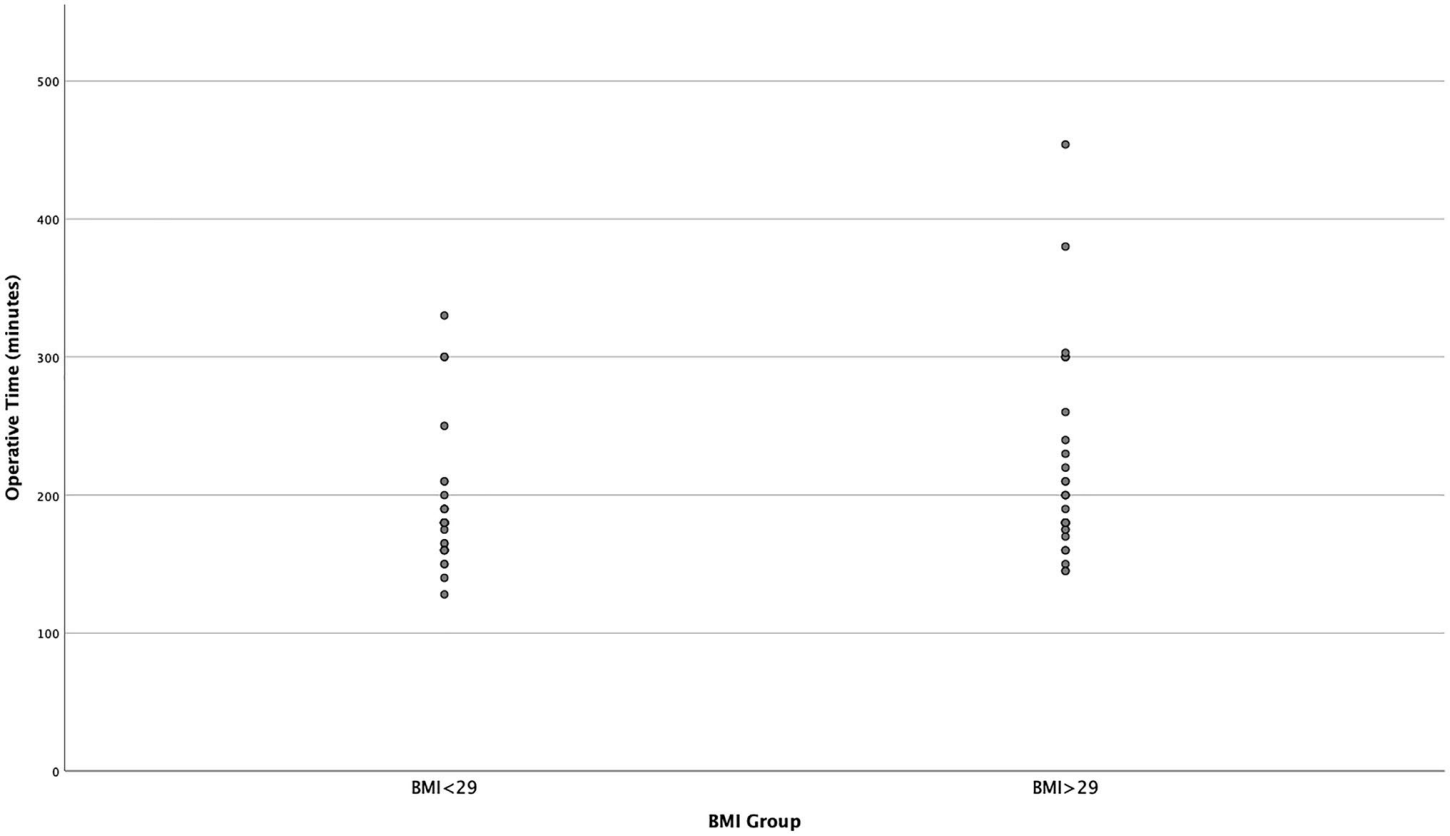
Graph comparing operative time in both groups (*p* = 0.05)

Median operative time was 186 vs. 216 min in groups 1 and 2, respectively (Fig. 3; *p* = 0.05).

**Fig. 4:**
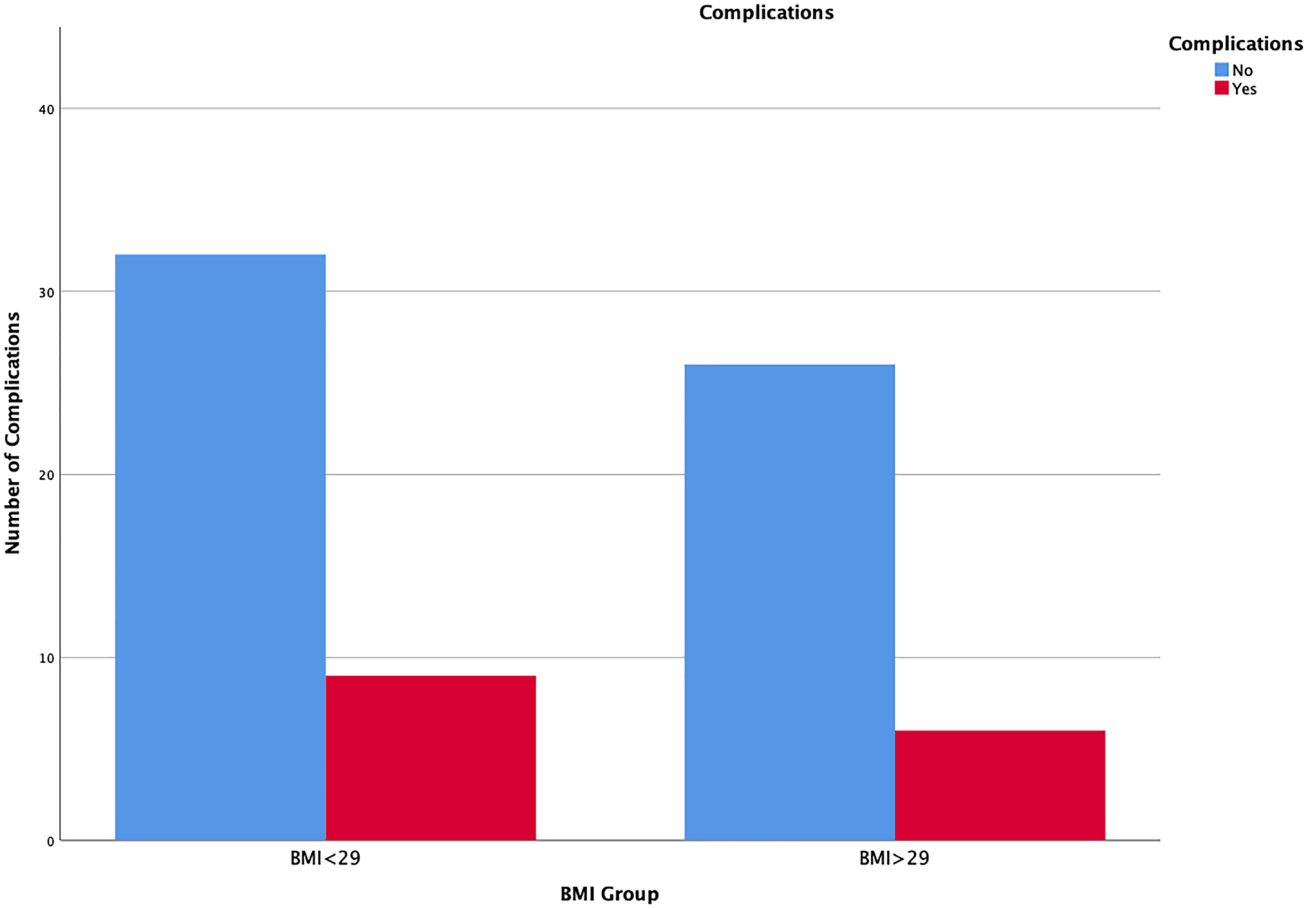
Boxplot illustrating overall complication ratio in both groups (*p* = 0.26)

Comparison of the overall complication rate was not significantly different in both groups with 8 (20%) in group 1 vs. 6 (19%) complications in group 2 (Fig. 4; *p* = 0.26). The distribution of group 1 vs. 2 included Clavien–Dindo grade I (2 vs. 3), Clavien–Dindo grade II (5 vs. 3) and Clavien–Dindo grade III (1 vs. 0). There was no Clavien–Dindo grade IV and above.

In group 1, there was one anastomotic leak, and one patient had a lymphatic leak causing a lymphocele managed conservatively. There was one patient with postoperative ileus in group 1 vs. 3 in group 2. There were 5 patients with wound infections in group 1 compared to 3 in group 2. There were no conversions in either group. There were no cases of major vascular injury. Average blood loss during surgery was 15 ml vs 10 ml in groups 1 and 2, respectively (*p* = 0.27).

Median LOS was 7 vs. 6 days in groups 1 and 2 (*p* = 0.48) and a similar number of patients were readmitted within 30 days (2 vs. 5 in groups 1 and 2, respectively; *p* = 0.13).

### Oncological outcomes

Oncological outcomes are listed in Table 2. There was no difference in pathologic margins with achievement of R0 resection in 98% and 94% in the two groups (*p* = 0.43). Median LNH was also found to be similar in both groups (31 vs. 30; *p* = 0.28) with LNR 0.06 vs. 0.08 (*p* = 0.49) in groups 1 and 2, respectively.

**Table 2:** Oncological outcomes

Parameter	Group 1 (*N* = 41)	Group 2 (*N* = 32)	*p* value
Positive resection margin R+ (*n*)	1	2	0.43
Lymph node harvest (*n*)	31	30	0.28
Lymph node positive (*n*)	16	16	0.51
Lymph node ratio	0.06	0.08	0.49
pTNM			
T (0/1/2/3/4)	1/8/24/8	2/8/18/4	0.70
N (0/1/2)	25/8/8	16/11/5	0.36
M (0/1)	37/4	30/2	0.37

## Discussion

Previously, our institution described the use of ultrasound to facilitate the vascular identification in CME surgery [10] and we have now evaluated postoperative outcomes after robotic CME, comparing non-obese (BMI ≤ 28 kg/m^2^) and obese (BMI > 29 kg/m^2^) patients, in which SMV detection was aided using the robotic ultrasound device. To our knowledge, this paper is the first to present the outcomes of novel use of robotic ultrasound for SMV identification.

Studies have shown that CME surgery in right sided colon cancers is associated with better oncological outcomes (lower recurrence and improved survival) compared to conventional surgery [1, 2]. This technically challenging procedure demands a careful dissection along the SMV, with associated risk of vascular injury, especially in obese patients where vascular structures are more difficult to identify intraoperatively.

The original concept of CME was proposed by Hohenberger in 2009 with lateral-to-medial mobilisation [2]. Since then, several approaches have been described, such as single port plus additional port CME and modification of the medial to lateral approach [11, 12]. A subileal approach was developed to facilitate laparoscopic CME, where central vascular ligation was carried out after mobilisation of the root of the mesocolon [13]. In obese patients, SMV first approach is an attractive option for primary vascular control [14]. In 2017, the suprapubic approach was reported, with port placement along a horizontal line 3–6 cm above the pubis [15, 16]. A standardized suprapubic bottom-up approach was then developed with caudolateral mobilisation of the right colon and subsequent dissection right of the middle supramesenteric vessels with central ileocolic vessel ligation [17]. In this study, a standardised CME with SMV first approach was used in which the first step was vessel dissection and ligation, and subsequent medial-to-lateral dissection.

Our data suggested that robotic CME resection was equally safe in non-obese and obese patients when USS was used in the latter group, with overall complication rate 20% vs. 19% in groups 1 and 2, respectively. Published literature on complication rate after CME report ranges between 19% [2] and 35.5% [17] postoperative complication rates and confirm the safety of CME and D3 dissections [18, 19]. There are, however, increased risks involved when performing a CME [20, 21] and surgeons should achieve proficiency by a training program and proctoring before taking on CME surgery. In addition, a learning curve must be taken into account for the use of rUSS. Literature concerning ultrasound training in other disciplines states that an average of 70–80 cases was required to obtain competence levels in soft-tissue scanning [22]. There is paucity of data on the learning curve for surgeons training in vascular ultrasound; however, from personal experience, vascular identification in TilePro vision was successfully achieved after 5 training cases. The learning curve to reach proficiency in robotic ultrasound scan should be explored in further studies.

Several studies have investigated the effect of obesity on surgical outcomes after colorectal resections. Benoist et al. found no difference in overall mortality or complication rate between obese and non-obese patients after left or right colectomy; however, they found significantly more postoperative intra-abdominal collections requiring treatment after left colectomy in obese patients (*p* < 0.05) and a significant higher leakage rate (*p* < 0.02) and mortality (*p* < 0.05) after proctectomy in obese patients [23]. A recent study on laparoscopic CME surgery showed the main complications to be Clavien–Dindo grades I and II, and elderly and obesity (BMI ≥ 28 kg/m^2^) are independent risk factors for postoperative complications [24]. Even though we did not find a significant difference in the number of wound infections, other studies have shown that BMI is incrementally associated with wound-related complications and slow healing in colonic surgery [25–27]. Our study shows that the obese patients recover as fast as non-obese patients with comparable mean LOS of 7 days in the non-obese group and 6 days in the obese group. A shorter length of stay was obtained in obese patients, possibly due to meticulous dissection and minimal morbidity.

The use of rUSS added 10 min [range 7–12 min] to the procedure; however, our results show that the median operative time differs almost significantly with a 30-min longer operative time in obese patients (Fig. 3; *p* = 0.05). This is in accordance with a study by Khoury et al., comparing complications in obese and non-obese patients after laparoscopic bowel resections, which shows a significantly increased mean duration of operation when performing a laparoscopic resection in obese patients compared to non-obese patients. (171.5 vs. 157.3 min; *p* = 0.017) [26]. When considering that in the previous study no USS was used and operative times were still significantly longer in obese patients, the prolonged operative time could be explained by the difficulty of the entire procedure due to adipose tissue with limited visibility and limited intra-abdominal space.

In addition, we found no difference in short term oncological outcomes. The lymph node ratio (LNR), the ratio of metastatic to total retrieved nodes, has shown to be a valuable prognostic factor in node-positive colon cancer [28]. Lower ratios (below 0.13) are associated with better survival. Our data showed R0 resection was achieved in 98% and 94% and here was no difference in lymph node harvest or LNR with D3 lymphadenectomy performed in both groups.

This study has some limitations. It is a single center retrospective study. There is no comparison to an obese group without the use of rUSS, only to literature complications and outcomes. Patients were enrolled using specific inclusion criteria; however, selection bias must be taken in account.

In conclusion, this study shows that the results after robotic CME in obese patients (BMI ≥ 29 kg/m^2^) are comparable to those of non-obese patients with longer operative times in obese patients due to technical challenges. Intraoperative ultrasound scan should be considered to facilitate robotic CME procedures to help identify the SMV and allow safer dissection in obese patients. Obesity is increasing globally and further research describing techniques to facilitate colorectal robotic procedures in obese patients are needed.

## Supplementary Information

Below is the link to the electronic supplementary material.

Supplementary file1 (DOCX 35 kb)

## Data Availability

Yes, anonymized data are available however patient privacy must be taken into account.
