# Supplementary material for: Robotic CME in obese patients: advantage of robotic ultrasound scan for vascular dissection
Source: J Robot Surg. 2022 Apr 15;17(1):155–61. doi: 10.1007/s11701-022-01398-6 (PMC9939489; doi:10.1007/s11701-022-01398-6)
Supplement: Supplementary file 1 — Supplementary file1 (DOCX 35 kb) [file 11701_2022_1398_MOESM1_ESM.docx]

# Supplementary Information

<https://doktervickymaertensalgemene-my.sharepoint.com/:v:/g/personal/admin_doktervickymaertensalgemene_onmicrosoft_com/EStoNvUVIUJIhlLssq3RbxQBMc8Nz662Uyf0JpP4kWZwEQ?e=wvkHws>

**Online Resource 1:** illustrating ultrasound view and marking of the SMV with subsequent vessel dissection along the superior mesenteric vein. SMV: superior mesenteric vein, ICA and ICV: ileocolic artery and vein, HT: Henle’s trunk, MCV: middle colic vein, PDV: pancreatic duodenal vein, LMCA: left branch of the middle colic artery, RMCA: right branch of the middle colic artery, AMCA: ascending branch of the middle colic artery.
